# Explainable AI for Image-Based Auxiliary Assessment of Radiation Pneumonitis on Post-Treatment CT Images Using GAN Grad-CAM and Ensemble Learning Techniques

**DOI:** 10.3390/life16081269

**Published:** 2026-07-31

**Authors:** Tsair-Fwu Lee, Guang-Zhi Lin, Wen-Ping Yun, Yi-Lun Liao, Liyun Chang, Chao-Hong Liu, Cheng-Shie Wuu, Yu-Chang Hu, Yu-Wei Lin, Pei-Ju Chao, Yang-Wei Hsieh

**Affiliations:** 1 Medical Physics and Informatics Laboratory of Electronic Engineering, National Kaohsiung University of Science and Technology, Kaohsiung 80778, Taiwan; tflee@nkust.edu.tw (T.-F.L.); haguy0507@gmail.com (G.-Z.L.); 1106405106@nkust.edu.tw (W.-P.Y.); frankliao60913@gmail.com (Y.-L.L.); 2 Graduate Institute of Clinical Medicine, Kaohsiung Medical University, Kaohsiung 80708, Taiwan; 3 Department of Medical Imaging and Radiological Sciences, Kaohsiung Medical University, Kaohsiung 80708, Taiwan; 4 Department of Medical Imaging and Radiological Sciences, I-Shou University, Kaohsiung 82445, Taiwan; cliyun2000@gmail.com; 5 Department of Dermatology, Kaohsiung Yuan’s General Hospital, Kaohsiung 80249, Taiwan; y4509@yuanhosp.com.tw; 6 Department of Radiation Oncology, Columbia University, New York, NY 10032, USA; csw6@columbia.edu; 7 Department of Radiation Oncology, Kaohsiung Veterans General Hospital, Kaohsiung 813414, Taiwan; ychu@vghks.gov.tw (Y.-C.H.); marklin1108@gmail.com (Y.-W.L.); 8 Department of Radiation Oncology, Kaohsiung Chang Gung Memorial Hospital and Chang Gung University College of Medicine, Kaohsiung 833401, Taiwan

**Keywords:** radiation pneumonitis, explainable artificial intelligence (explainable AI), generative adversarial network (GAN), Grad-CAM, ensemble learning

## Abstract

**Objective:** This study aims to develop a preliminary explainable image-based auxiliary assessment framework for radiation pneumonitis (RP) by integrating a generative adversarial network (GAN), Gradient-weighted Class Activation Mapping (Grad-CAM), and ensemble learning to improve image-based classification performance, interpretability, and computational efficiency. **Methods:** Chest CT images from 46 lung cancer patients who underwent VMAT were retrospectively collected, yielding 542 RP and 1857 non-RP images. Images were preprocessed using Otsu-based segmentation and standardized before being input into the RP-GAN for feature extraction. Grad-CAM was applied to qualitatively visualize attention patterns across convolutional layers and guide feature-layer selection, while PCA reduced the extracted features from 12,288 to 730 dimensions. An ensemble stacking classifier combining RF, SVM, KNN, and XGBoost with logistic regression as a meta-learner was constructed. Model performance was evaluated using AUC, accuracy, PPV, NPV, specificity, recall, and F1-score. **Results:** Grad-CAM highlighted conv2d_4 and conv2d_5 as the most informative layers. PCA reduced training time from 49 min to 49 s with minimal performance loss. In the internal hold-out test set, the ensemble model achieved the highest point estimates for AUC and accuracy among the evaluated classifiers (AUC = 0.921; accuracy = 87.1%). The model identified RP-positive CT slices and provided qualitative visual cues for suspected RP-related regions. **Conclusions:** The proposed GAN–Grad-CAM ensemble framework showed promising internal classification performance for post-treatment CT-based RP image assessment with substantially improved computational efficiency. Its qualitative visual outputs may support clinical image review, although further external validation and quantitative localization assessment are required before clinical application as an auxiliary image-review tool.

## 1. Introduction

Radiation pneumonitis (RP) is a common and potentially serious complication following radiotherapy for lung cancer. Recent systematic review and meta-analysis evidence has further emphasized that RP remains an important post-radiotherapy complication and that machine learning-based models using multimodal features may provide useful support for RP risk prediction in lung cancer patients [1]. However, image-only models cannot directly incorporate dosimetric and clinical factors, such as radiation dose distribution, pulmonary function, treatment history, and patient-specific characteristics, which may limit comprehensive RP assessment and the differentiation of RP-related changes from other pulmonary abnormalities. Accordingly, the present study does not aim to develop a comprehensive RP risk-prediction model but instead focuses on the preliminary evaluation of an explainable image-based auxiliary review framework using post-treatment CT images. Clinical manifestations include coughing and shortness of breath, and in severe cases, RP may necessitate corticosteroid treatment, oxygen therapy, hospitalization, or even result in mortality.

The diagnosis of RP is often challenging due to subtle and non-specific imaging features, coupled with variability stemming from patient characteristics, treatment modalities, and dose distributions. Traditional diagnostic approaches primarily depend on manual interpretation of CT images by clinicians, a process that is inherently subjective and susceptible to missed diagnoses. Therefore, there is a need for objective and efficient image-based auxiliary tools that can support, rather than replace, clinician review.

Artificial intelligence (AI), especially deep learning models, has shown promise in medical image analysis but is often criticized for its “black box” nature. Grad-CAM helps address this by visualizing model attention and revealing decision mechanisms [2,3]. Yoo et al. [4] showed that Grad-CAM effectively identifies key CNN layers, improving interpretability, while Zou et al. [5] demonstrated its ability to highlight clinically relevant regions in RP prediction tasks.

Despite recent advances in convolutional neural networks (CNNs) [6,7] for medical imaging, key limitations remain. These include poor interpretability, limited accuracy in complex cases, and low computational efficiency, all of which negatively affect clinical adoption [3,6,7]. Moreover, many existing studies rely on single-model frameworks, fail to address feature redundancy in high-dimensional imaging data, and are not optimized for clinical usability.

Recent image-based studies of radiation pneumonitis have mainly focused on pre-treatment risk prediction using different deep learning or radiomics approaches. Kapoor et al. [8] applied a three-dimensional deep convolutional neural network to pre-treatment CT images, whereas Zhang et al. [9] developed a deep learning model integrating CT and radiation dose images. Lee et al. [10] combined pre-treatment chest CT images with clinical factors, and Zou et al. [5] employed a CNN–RNN framework with Grad-CAM analysis. Radiomics-based approaches have also been investigated for RP prediction [11], while Ninomiya et al. [12] used Betti number maps for RP risk stratification. Overall, these studies primarily addressed pre-treatment RP risk prediction using supervised CNN-based, multimodal, or handcrafted-feature approaches. In contrast, the present study focuses on post-treatment CT-based RP image assessment and uses the discriminator of an RP-GAN for unsupervised feature representation, followed by Grad-CAM visualization, PCA dimensionality reduction, and stacking ensemble classification. This integrated framework was designed to jointly address image-based classification performance, interpretability, and computational efficiency.

To address these gaps, we propose a preliminary image-based auxiliary assessment framework for post-treatment RP image assessment. Our approach integrates four complementary techniques:

Generative Adversarial Networks (GAN) for effective feature extraction, Grad-CAM for interpretability, Principal Component Analysis (PCA) for dimensionality reduction, and ensemble learning for robust classification. While each of these techniques has been individually used in prior studies, to the best of our knowledge, their integration into a unified framework for RP image assessment has not been previously explored.

Our proposed model—Radiation Pneumonitis GAN (RP-GAN) is designed to address three major challenges in image-based RP assessment: (1) variability in image-based classification performance due to subjective image interpretation, (2) lack of interpretability in deep learning models, and (3) inefficiencies associated with high-dimensional medical image data. We leverage the discriminator of the GAN to extract high-level semantic features through unsupervised learning, which may be useful for tasks with limited annotated medical data, as it allows the model to learn image features related to RP lesions without manual labeling. These features are then compressed using PCA [13] to reduce dimensionality and accelerate training without compromising accuracy, while the ensemble classifier architecture (based on stacking RF, SVM, KNN, and XGB with logistic regression as a meta-learner) improves classification stability and generalizability.

This study presents an interpretable and computationally efficient image-based framework for RP classification. The proposed RP-GAN framework provides classification outputs and qualitative visual cues that may support clinical image review. By integrating GAN-based feature extraction, Grad-CAM visualization, PCA dimensionality reduction, and stacking ensemble learning, this study aims to balance image-based classification performance, interpretability, and computational efficiency in a unified RP image-analysis pipeline.

## 2. Materials and Methods

### 2.1. Research Framework

**Figure 1:**
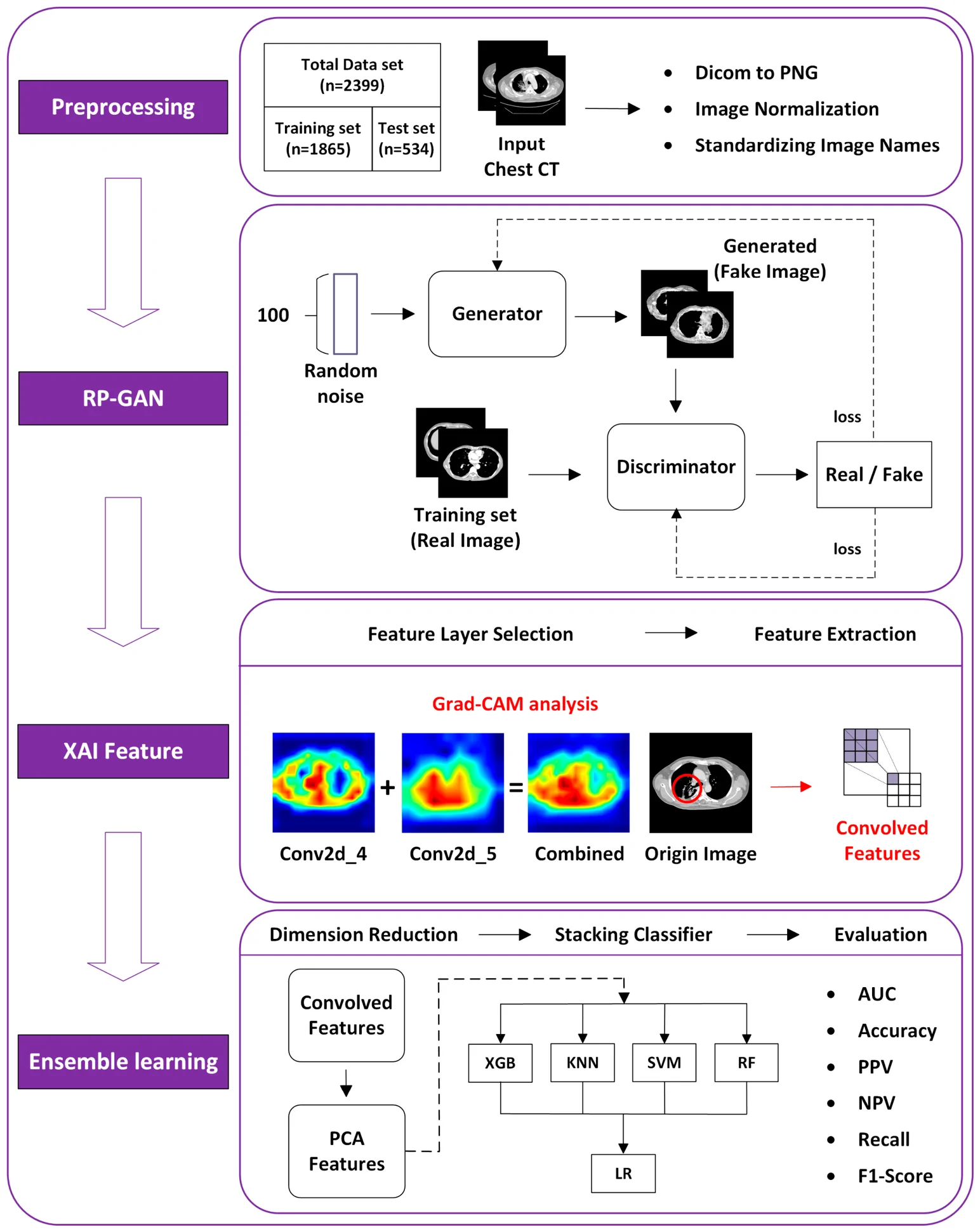
Overall research flowchart, including data preprocessing, RP-GAN model training, XAI feature extraction, and ensemble learning model construction and evaluation. Abbreviations: CT: Computed Tomography, DICOM: Digital Imaging and Communications in Medicine, PNG: Portable Network Graphics, RP-GAN: Radiation Pneumonitis Generative Adversarial Network, XAI: Explainable Artificial Intelligence, PCA: Principal Component Analysis, XGB: Extreme Gradient Boosting, KNN: K**-**Nearest Neighbor, SVM: Support Vector Machine, RF: Random Forest, LR: Logistic Regression, AUC: Area Under the ROC Curve, ROC: Receiver Operating Characteristic, PPV: Positive Predictive Value, NPV: Negative Predictive Value.

This study aims to establish a preliminary image-based auxiliary review framework for RP assessment on post-treatment chest CT images, as illustrated in Figure 1. Initially, the collected patient CT images are preprocessed. The training set is subsequently input into the modified RP-GAN model to train the generative adversarial network. During the training process, Grad-CAM technology is utilized to qualitatively visualize attention patterns across convolutional layers and guide feature-layer selection according to predefined qualitative criteria, including attention within the thoracic and lung regions, avoidance of excessive activation in non-clinically relevant background structures, and visual consistency with suspected RP-related CT findings. No quantitative activation threshold or layer-wise contribution cutoff was applied. Finally, the extracted image features are input into a stacking classifier, which integrates the predictive results of multiple classifiers to construct a more stable image-based classification model, and its performance is comprehensively evaluated using various metrics.

This study employs five main techniques: Otsu thresholding, RP-GAN generative adversarial networks, Grad-CAM, PCA, and ensemble learning. Specifically, the Otsu method is used to eliminate nontarget areas in the images, reducing noise interference; RP-GAN leverages generative and discriminative adversarial learning to extract important features from CT images; Grad-CAM technology visualizes the activation layers of CNNs, displaying their contribution to the model’s predictions; PCA efficiently filters representative features and performs dimensionality reduction to increase the model’s computational efficiency; and ensemble learning combines multiple models to reduce the risk of overfitting in a single model and improve generalizability.

### 2.2. Data Collection

This study retrospectively collected chest CT images obtained 3–6 months after VMAT from lung cancer patients treated at Kaohsiung Veterans General Hospital (KSVGH) between 2018 and 2021. The images were captured via a Toshiba-Aquilion computed tomography scanner, and all the images were saved in a 512 × 512 pixel, 16-bit Digital Imaging and Communications in Medicine (DICOM) format, excluding those CT images with contrast agent injections. The use of patient data in this study was approved by the Institutional Review Board (IRB) of Kaohsiung Veterans General Hospital, with the approval numbers KSVGH23-CT12-03 and KSVGH25-CT1-06. This study strictly adhered to ethical standards and regulatory requirements. Given the retrospective study design and the use of de-identified clinical and imaging data, with no additional intervention, treatment modification, or direct patient risk, the requirement for written informed consent was formally waived by the Institutional Review Board.

Table 1 summarizes the baseline clinical characteristics of the 46 lung cancer patients enrolled in this study, including age, gender, BMI, tumor staging (T and N stages), and whether chemotherapy was administered. These characteristics are provided for descriptive purposes only and were not used as input features for model training.

**Figure 2:**
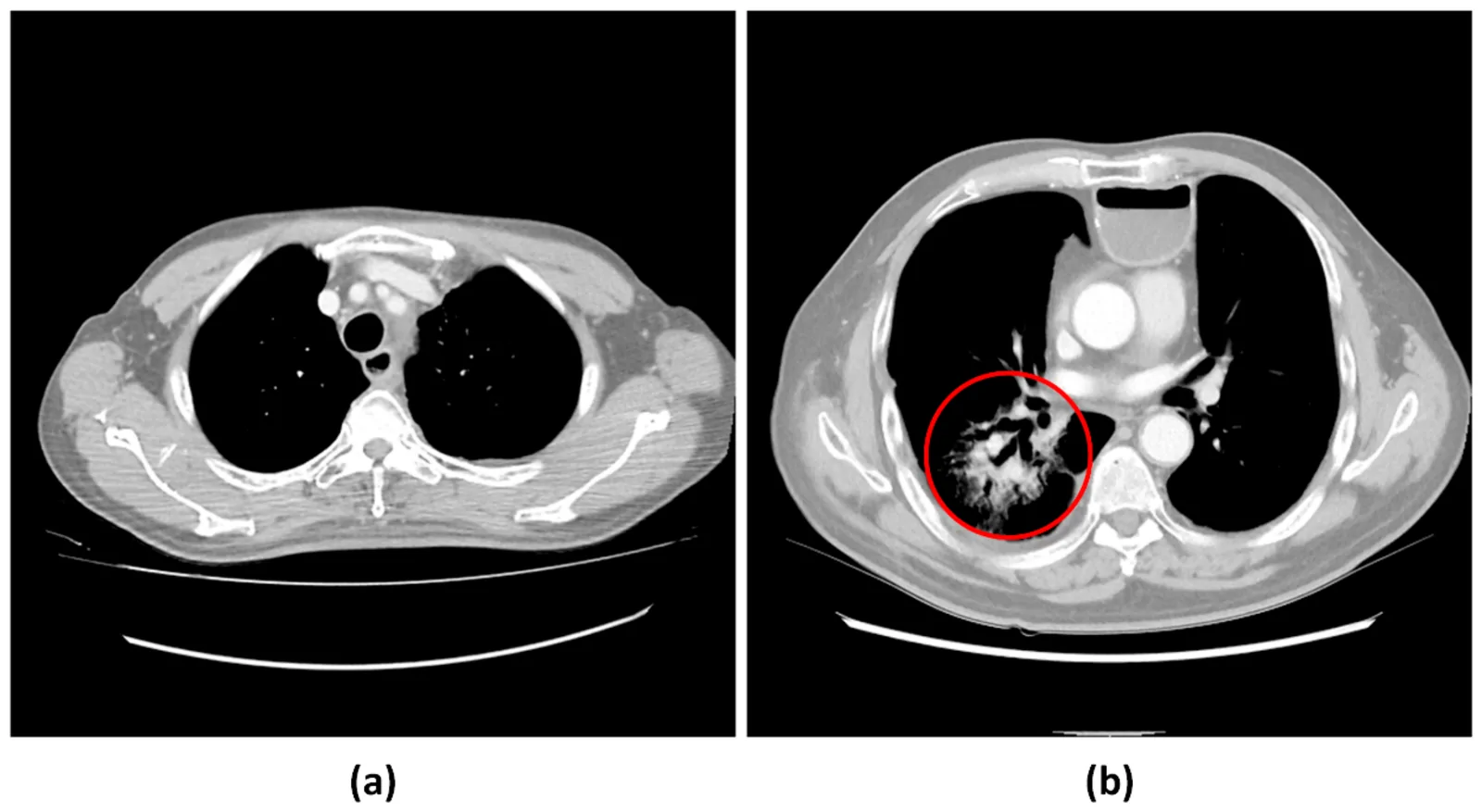
(**a**) Normal CT image and (**b**) RP CT image, with the red circle indicating a suspected RP-related region for qualitative visual reference. Abbreviations: CT: Computed Tomography, RP: Radiation Pneumonitis.

RP status was retrospectively determined by two radiation oncologists based on chest CT findings and clinical manifestations. The diagnostic criteria were as follows according to the Radiation Therapy Oncology Group (RTOG) radiation pneumonitis grading system: Grade 0 indicates no symptoms; Grade 1 indicates mild symptoms, usually with minor changes observable via medical imaging, and the patient may experience mild coughing or breathing difficulties; Grade 2 indicates moderate symptoms, possibly with persistent coughing or breathing difficulties requiring oral medication; Grade 3 indicates severe symptoms, potentially with aggravated breathing difficulties or persistent chest pain needing oxygen therapy; and Grade 4 indicates very severe symptoms, life-threatening and requiring hospitalization, possibly involving the need for assisted ventilation. The RP category included patients with any signs of RP in their CT images (≥Grade 1), while all others were classified as non-RP. We retrospectively collected CT images from 46 lung cancer patients (35 with RP and 11 with non-RP) who underwent VMAT 3–6 months post-treatment at Kaohsiung Veterans General Hospital (2018–2021), yielding a total of 542 RP images and 1857 non-RP images. The cohort size was determined by the availability of all eligible patients with complete post-treatment CT data during the study period, rather than by a prospective sample size or statistical power calculation. Therefore, the present cohort should be regarded as a preliminary feasibility sample. Although multiple CT slices were available from each patient, the effective clinical sample size remained 46 patients, including only 11 non-RP cases. The dataset was divided into training and test sets at an approximately 8:2 ratio at the patient level rather than the slice level. All CT slices from the same patient were assigned exclusively to either the training set or the test set, ensuring that no patient contributed slices to both subsets and minimizing the risk of data leakage. The dataset for this study is shown in Table 2. Figure 2 shows the two most representative clinical image cases in this study. Figure 2a is a non-RP image, which displays a normal lung structure with uniform lung fields and no abnormal shadows or lesions. Figure 2b is an RP image, showing typical features of RP, such as ground-glass opacities.

### 2.3. Data Preprocessing

In the data preprocessing phase, we adopted the following steps to ensure data consistency and increase the stability of subsequent deep learning model training:

**Format Conversion:** We converted the original DICOM images into PNG format. This step preserves the original quality of the images, avoids information loss due to compression, and facilitates subsequent image processing and model training.

**Image Segmentation and Centering:**

We employed an adaptive segmentation method that combines Otsu thresholding and contour analysis. Otsu thresholding was selected because it automatically determines an image-specific threshold based on the grayscale intensity distribution, without requiring manual threshold specification, and has been used in CT-based lung image preprocessing and GAN-related lung image segmentation frameworks [14]. In this procedure, each grayscale CT slice was first processed using Otsu thresholding to generate a binary image separating foreground structures from the background. Contour detection was then applied to the binary image to identify all connected foreground regions. Among the detected contours, the contour with the largest area was selected as the main thoracic region, and a binary mask was generated from this contour. The mask was subsequently applied to the original CT slice to retain the thoracic region while suppressing non-clinically relevant background structures, such as the diagnostic bed and peripheral non-body regions. This preprocessing step was intended to guide the model to focus on the thoracic cavity rather than to perform fine lesion segmentation. After masking, image centering was performed to reduce positional variation in the retained thoracic region across CT slices. All 2399 CT slices were visually inspected after preprocessing to confirm that the main thoracic region was consistently retained and that no gross segmentation failure occurred during quality review. The preprocessing pipeline did not include a dedicated automated correction mechanism for severe anatomical distortion or surgical hardware artifacts; therefore, visual quality control was used to verify that the main thoracic region was adequately retained in each processed slice. Although no gross masking failure was identified in the present cohort, subtle inconsistencies associated with distorted anatomy or high-density hardware artifacts could not be completely excluded.

**Data Standardization:** The processed PNG images are standardized in pixel values, adjusting their range to the interval [−1, 1] to enhance consistency between data points.

### 2.4. RP-GAN Feature Extraction Model Development

**Figure 3:**
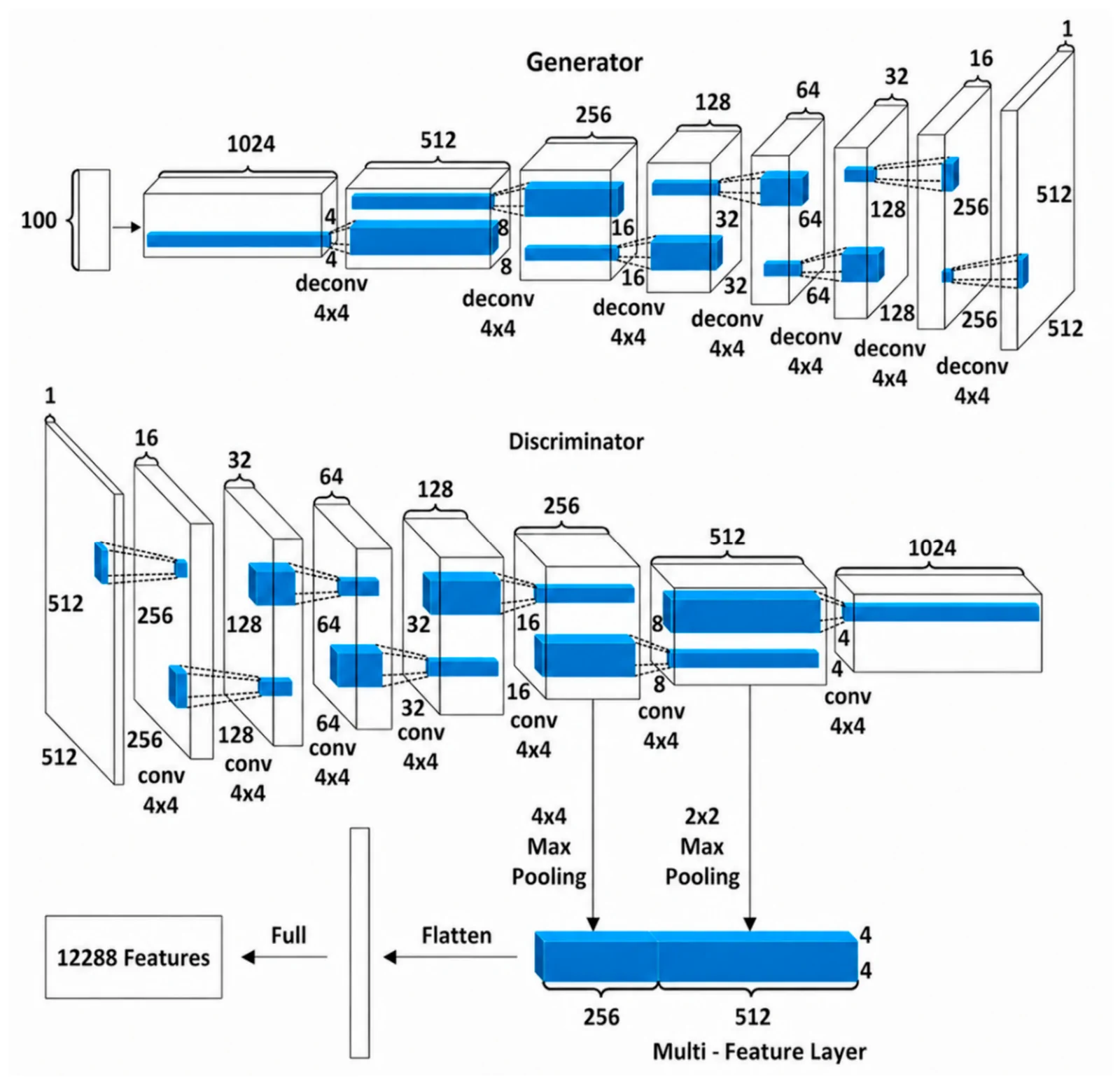
The RP-GAN architecture used for feature extraction in this study. The generator produces synthetic grayscale CT-like images from a 100-dimensional random noise vector, whereas the discriminator learns hierarchical image representations through seven convolutional layers. In this study, the pooled outputs of conv2d_4 and conv2d_5 were flattened and concatenated to form a 12,288-dimensional feature vector for subsequent PCA dimensionality reduction and stacking classification. Abbreviations: deconv: deconvolution, conv: convolution, RP-GAN: Radiation Pneumonitis Generative Adversarial Network.

This study modifies the Lung-GANs architecture proposed by Yadav et al. [15] to accommodate grayscale images. Lung-GANs was selected because it was originally developed for representation learning in lung CT and chest imaging tasks, which is closely aligned with the imaging domain of this study. In addition, its discriminator can extract high-level image features without directly relying on RP/non-RP class labels during the feature learning stage, making it suitable for medical imaging studies with limited annotated data. All model development experiments were conducted using Python 3.10 and TensorFlow 2.0 with CUDA 11.2 on a workstation equipped with an NVIDIA GeForce RTX 4070 Ti Super GPU with 16 GB of memory. The hyperparameters for the training process were set as follows: batch_size = 32, epochs = 100, and both the generator and discriminator used the Adam optimizer with a learning rate of 2 × 10^−4^ and beta_1 of 0.5. These hyperparameters were selected based on the original Lung-GAN architecture and commonly used GAN training settings, without adding additional hyperparameter optimization procedures. Specifically, a batch size of 32 was used to balance GPU memory usage, training efficiency, and gradient stability, while 100 epochs were adopted to provide sufficient training iterations for stable feature learning. No systematic hyperparameter search, such as grid search or random search, was performed in this study; future studies will further evaluate the effect of hyperparameter optimization on model performance and stability. The RP-GAN feature extraction architecture is illustrated in Figure 3 and is detailed below.

### 2.5. Grad-CAM Visualization Analysis

This study employs Grad-CAM technology, a widely used method in the field of XAI, to generate heatmaps that trace the contribution of each convolutional layer in the discriminator to the predicted class, highlighting regions associated with model attention. This visualization facilitates the identification of the most suitable convolutional layers as feature extraction layers. For layer selection, Grad-CAM heatmaps were generated for all convolutional layers in the RP-GAN discriminator, including conv2d through conv2d_6, and were compared qualitatively. The comparison focused on whether the attention maps were mainly distributed within the thoracic and lung regions, whether excessive activation in non-clinically relevant background structures was avoided, and whether the high-activation regions were visually consistent with suspected RP-related image findings on CT images. Earlier convolutional layers tended to capture low-level structural and texture-related information and produced relatively diffuse attention maps. From an architectural perspective, each convolutional layer in the discriminator uses a stride of 2; therefore, the spatial resolution progressively decreases, while the channel depth and level of feature abstraction increase as the network becomes deeper. Before the subsequent pooling operations, conv2d_4 and conv2d_5 generate feature maps of 16 × 16 × 256 and 8 × 8 × 512, respectively. Compared with the earlier layers, these intermediate-to-deep layers encode more abstract semantic and texture representations while retaining more regional spatial information than the final conv2d_6 layer. This balance between spatial resolution and feature abstraction may facilitate the representation of regional pulmonary patterns, including patchy parenchymal density changes and ground-glass-like abnormalities. Consistent with this architectural interpretation, conv2d_4 and conv2d_5 showed more visually informative and clinically relevant attention patterns and were therefore selected as the feature extraction layers. However, this interpretation does not indicate that these layers directly correspond to specific biological mechanisms of RP. The selection was based on predefined qualitative Grad-CAM assessment criteria rather than a quantitative activation threshold or layer-wise contribution cutoff. The Grad-CAM heatmaps were calculated as follows. Let the activation tensor of a selected convolutional layer be denoted as $A\in R^{H\;\times \;W\;\times \;K}\;$ where *H* and *W* represent the spatial dimensions of the feature maps and *K* represents the number of channels. The activation map of the *k*-th channel is denoted as $A^{k}\in R^{H\;\times \;W}$. For the target output $y^{c}$, the gradient $\frac{\partial y^{c}}{\partial A_{ij}^{k}}$ was calculated at each spatial location $\left(i,j\right)$. Global average pooling was then applied across the *H* × *W* spatial dimensions to obtain one scalar importance weight, $\alpha_{k}^{c}$, for each feature-map channel:

(1) $$\alpha_{k}^{C}=\frac{1}{Z}\sum_{i}\sum_{j}\frac{\partial y^{C}}{\partial A_{\mathrm{i}j}^{k}}$$

where *Z = H × W* represents the total number of spatial locations in the feature map. Accordingly, each $\alpha_{k}^{c}$ is a scalar, and the complete Grad-CAM weight vector $\alpha^{c}$ has dimension *K*. The final Grad-CAM heatmap was generated by calculating the weighted sum of the *K* activation maps and applying the ReLU function to retain only features that positively contributed to the target output:

(2) $$L_{Grad-CAM}^{c}=\;ReLU(\sum_{k=1}^{K}\alpha_{k}^{c}A^{k})$$

The resulting Grad-CAM heatmap $L_{Grad-CAM}^{c}$ has a spatial dimension of *H* × *W*. Based on the activation tensor dimensions described above, the complete weight vectors for conv2d_4 and conv2d_5 contained 256 and 512 scalar weights, respectively, and the corresponding heatmaps had spatial dimensions of 16 × 16 and 8 × 8 before resizing. The heatmaps were subsequently resized to the original CT image size of 512 × 512 for visualization and image overlay.

### 2.6. Generator Architecture

The generator receives input from a 100-dimensional random noise vector, which is mapped through a fully connected layer into a 4 × 4 × 1024 high-dimensional tensor. After batch normalization and ReLU activation, this tensor is reshaped into a three-dimensional tensor. It then enters the deconvolution phase, comprising six deconvolution layers each using 4 × 4 convolutional kernels, a stride of 2, and ‘same’ padding, with the number of filters progressively set at 512, 256, 128, 64, 32, and 16. Except for the final layer, each layer is equipped with batch normalization (momentum 0.9) and a ReLU activation function, gradually enlarging the feature map to a size of 512 × 512 × 1.

### 2.7. Discriminator Architecture

The discriminator consists of seven convolutional layers, each utilizing 5 × 5 convolutional kernels, a stride of 2, and ‘same’ padding. The first layer contains 16 filters, with subsequent layers increasing the number of filters to 32, 64, 128, 256, 512, and 1024. Except for the first layer, each layer is followed by batch normalization and a LeakyReLU activation function (α = 0.2). The output of conv2d_4 was reduced using 4 × 4 max pooling, resulting in a 4 × 4 × 256 feature tensor, whereas the output of conv2d_5 was reduced using 2 × 2 max pooling, resulting in a 4 × 4 × 512 feature tensor. These two tensors were flattened and concatenated to form a 12,288-dimensional feature vector for subsequent PCA dimensionality reduction and classification. Although conv2d_6 produced a 4 × 4 × 1024 output, this representation was not included in the PCA or downstream classification procedures. Instead, during adversarial training, the conv2d_6 output was separately passed to the discriminator’s fully connected output layer to generate a single authenticity score.

In our Keras implementation, these seven convolutional layers are automatically named “conv2d” (for the first layer) and “conv2d_1” through “conv2d_6” (for the second to seventh layers), respectively.

### 2.8. PCA Feature Dimensionality Reduction

This study employs the PCA method to reduce the dimensionality of features, enhancing the model’s computational efficiency and reducing multicollinearity among features. Initially, the feature data are standardized to ensure that each feature has a mean of 0 and a standard deviation of 1. This helps prevent variables with larger feature values from excessively influencing the PCA results. After standardization, PCA is applied to reduce the dimensionality of the data, selecting the number of principal components that can explain 95% of the data variance. PCA calculates the eigenvalues and eigenvectors and orders them by the size of the eigenvalues, choosing the eigenvectors corresponding to the top k eigenvalues as the principal components. The chosen number of principal components, k, ensures that the selected principal components can explain more than 95% of the data variance. In the present dataset, this criterion reduced the original 12,288-dimensional feature vector to 730 principal components, which together explained at least 95% of the cumulative variance.

### 2.9. Ensemble Learning—Stacking Classifier

**Figure 4:**
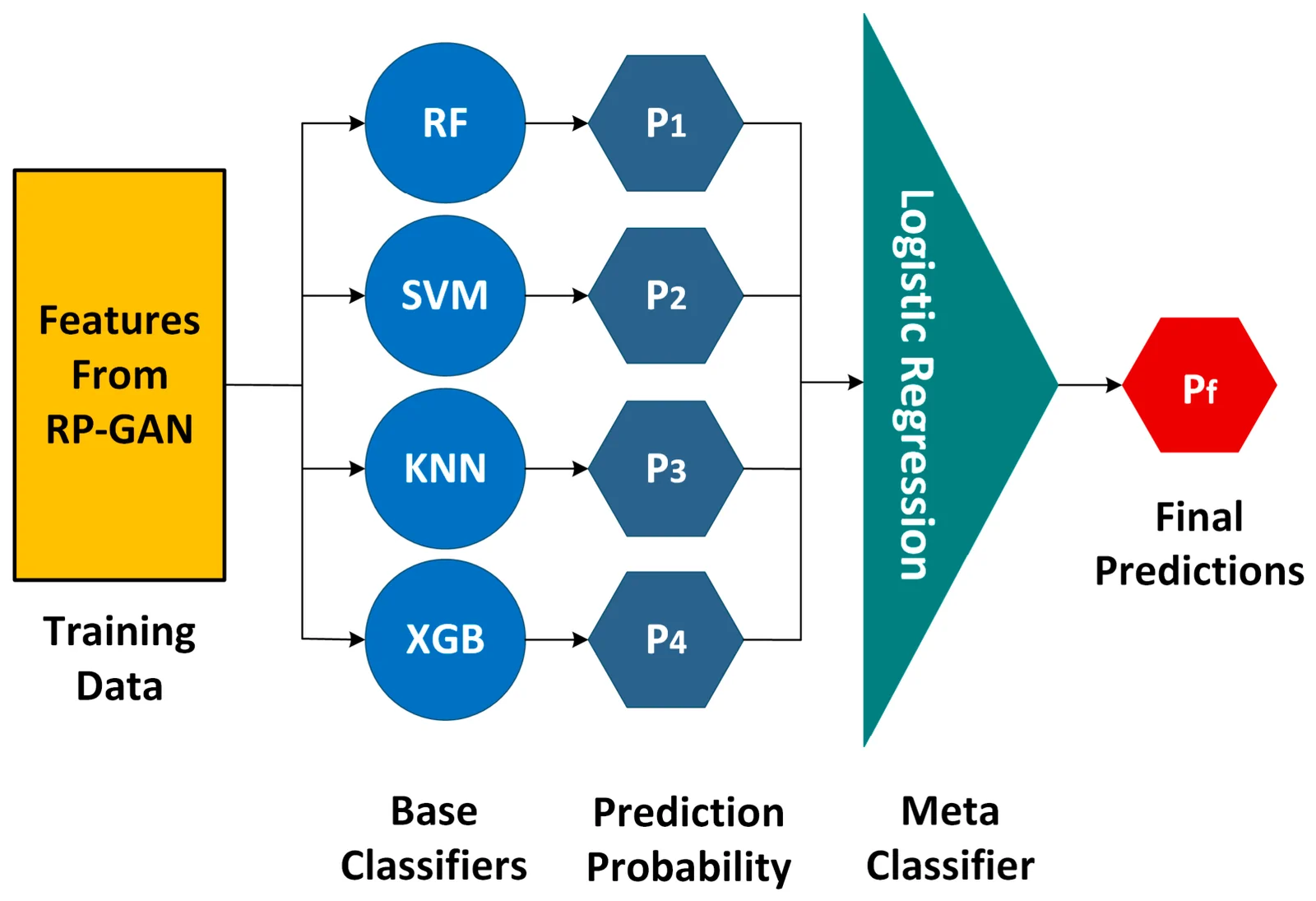
Stacking classifier architecture. Abbreviations: RP-GAN: Radiation Pneumonitis Generative Adversarial Network, RF: Random Forest, SVM: Support Vector Machine, KNN: K-Nearest Neighbor, XGB: Extreme Gradient Boosting.

This study adopts the stacking classifier method from ensemble learning, which combines multiple base classifiers and a meta-classifier. The selected base classifiers include random forest (RF), support vector machine (SVM), k-nearest neighbors (KNN), and extreme gradient boosting (XGB), with logistic regression (LR) ultimately used as the meta-classifier. These base classifiers were selected to provide complementary learning mechanisms for the high-dimensional RP-GAN-derived image features. RF was included because, as a tree-based ensemble method, it can handle nonlinear feature interactions and high-dimensional data while reducing overfitting risk. SVM was selected for its strong generalization ability in high-dimensional and nonlinear classification tasks, making it suitable for limited medical imaging datasets. KNN was included as a non-parametric classifier that can capture local similarity patterns among feature representations. XGB was selected because gradient boosting can iteratively reduce prediction errors and model complex feature interactions. LR was used as the meta-classifier because it provides a simple and interpretable way to integrate the prediction outputs of the base classifiers while limiting additional model complexity. Therefore, the selected classifiers were intended to increase model diversity and combine complementary decision mechanisms within the stacking framework. This approach enhances the overall prediction performance by integrating the predictive results of multiple models. The architecture of the stacking classifier is illustrated in Figure 4.

In the first layer, the base classifiers (RF, SVM, KNN, and XGB) were trained using only the predefined training set. Tenfold cross-validation was performed exclusively within the training set to generate out-of-fold predictions from each base classifier. Specifically, the training set was divided into ten folds. In each iteration, nine folds were used to train the base classifiers, while the remaining fold was used to generate validation predictions. This procedure was repeated ten times so that each fold served as the validation fold once. The resulting out-of-fold predictions from the four base classifiers were combined to form the meta-level training feature vectors. The internal hold-out test set was not included in any cross-validation fold and was strictly excluded from base-classifier training, generation of meta-level training features, and meta-classifier training.

These out-of-fold prediction vectors were used to train the second-layer meta-classifier (LR). After completion of the stacking model development process, the internal hold-out test set was used only for final independent performance evaluation. Through the stacking classifier technique, we are able to leverage the strengths of various models fully, enhance the model’s generalization ability across different datasets, and ultimately improve the accuracy and stability of the classification of RP.

During the training phase of the base classifiers, model-level class weighting strategies were applied to mitigate the potential bias introduced by class imbalance. RF and SVM employed the parameter setting class_weight = ‘balanced’, while XGBoost was configured with scale_pos_weight = 3. These adjustments were used to reduce the tendency of the classifiers to favor the majority class, rather than to fully resolve the underlying imbalance in patient-level sample distribution. Since KNN does not support class weighting, no such adjustment was applied; however, its potential bias can be compensated by the ensemble strategy through the outputs of other classifiers, contributing to the overall balance and stability of model performance. The meta-classifier (LR) uses the prediction outcomes from the first-layer classifiers as input, which represent a transformed feature space, and thus no additional class balancing adjustments were made at this stage.

### 2.10. Model Evaluation Methods

To evaluate the effectiveness of the proposed machine learning model, this study uses metrics such as the area under the curve (AUC), accuracy, positive predictive value (PPV), negative predictive value (NPV), specificity, recall, and F1-score. These evaluation metrics are derived from calculations based on true positives (TPs), true negatives (TNs), false positives (FPs), and false negatives (FNs) in the model’s prediction results, providing a comprehensive examination of the model’s performance in various aspects.

Additionally, to provide a more comprehensive assessment of the model’s performance under class imbalance, this study also incorporates the precision–recall (PR) curve and its associated average precision (AP) as evaluation metrics, further enhancing the analysis of the model’s performance in detecting positive samples.

## 3. Results

### 3.1. Grad-CAM Analysis

**Figure 5:**
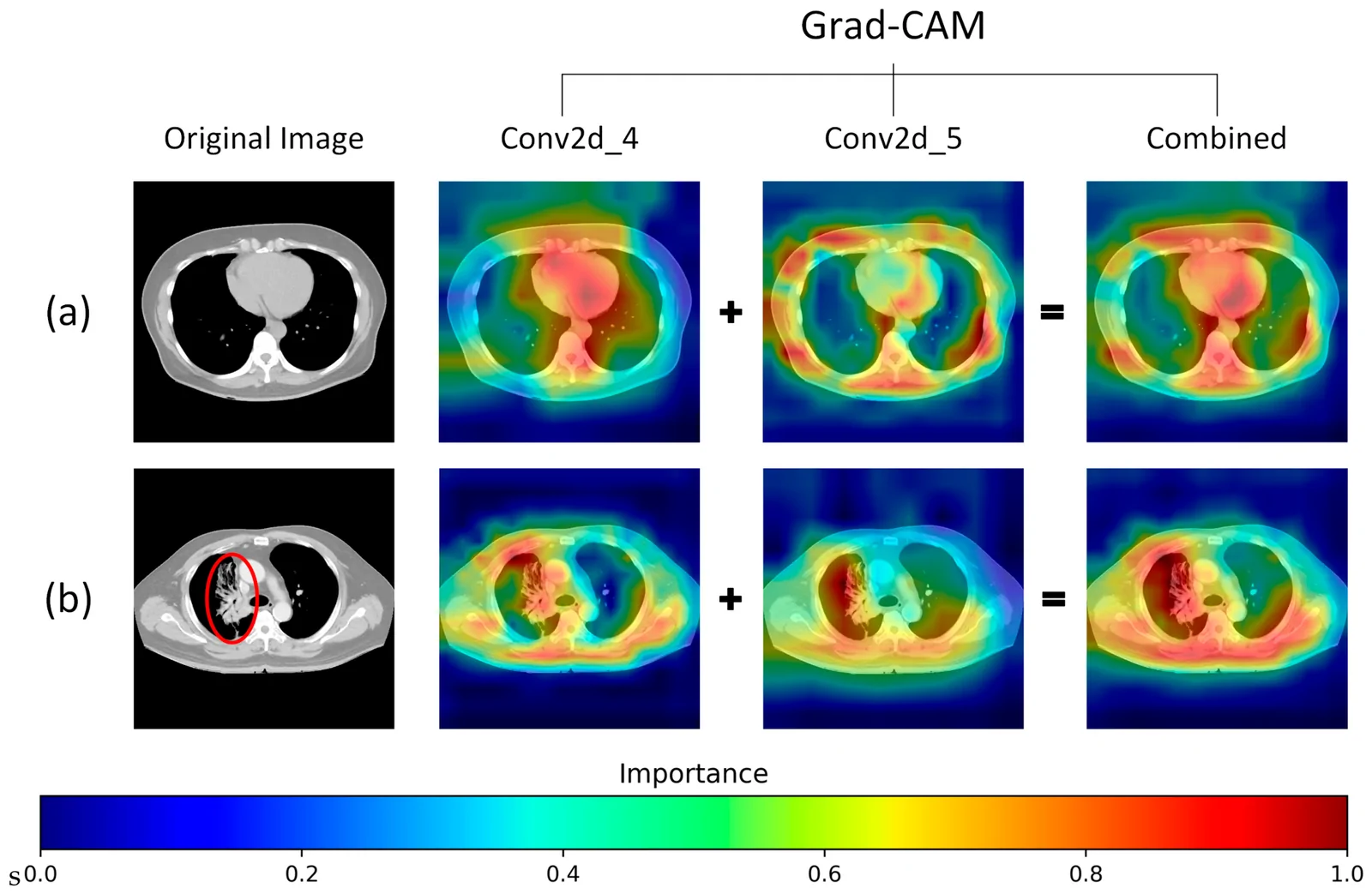
Heatmaps generated using Grad-CAM technology, providing a visual analysis for the two categories: (**a**) Non-RP and (**b**) RP, with the red circle indicating a suspected RP-related region for qualitative visual reference. Abbreviation: Grad-CAM: Gradient-weighted Class Activation Mapping, RP: Radiation Pneumonitis.

Grad-CAM heatmaps were generated for all convolutional layers of the RP-GAN discriminator, from conv2d to conv2d_6, and were qualitatively compared to evaluate layer-wise attention patterns. Based on visual assessment of the Grad-CAM heatmaps, conv2d_4 and conv2d_5 showed more clinically relevant and visually informative attention patterns for RP-related regions and were therefore selected for subsequent feature extraction, as shown in Figure 5. After visual analysis of the Grad-CAM heatmaps for the non-RP and RP images was performed, different attention distributions were observed between the two classes. In Figure 5a, the high-attention regions for non-RP images were mainly concentrated around the lung periphery, whereas in Figure 5b, the high-attention regions for RP images extended to areas visually consistent with RP-related findings. These results suggest that conv2d_4 and conv2d_5 may provide visually meaningful feature representations for RP classification. However, this interpretation is based on qualitative heatmap assessment rather than quantitative layer-wise contribution analysis.

To further evaluate the interpretability of the model, the selected convolutional layers (conv2d_4 and conv2d_5) were compared using different visualization techniques, including Grad-CAM++, SmoothGrad, and LayerCAM, as shown in Supplementary Figure S1. Qualitative observations indicated that Grad-CAM, Grad-CAM++, SmoothGrad, and LayerCAM generated high-attention regions that were visually consistent with suspected RP-related imaging findings. Among them, Grad-CAM, Grad-CAM++, and SmoothGrad produced heatmaps with higher intensity, predominantly focused on suspected RP-related regions and extending into surrounding structures, indicating broader regions of model attention. In contrast, LayerCAM produced a more concentrated heatmap specifically around visually suspected RP-related regions, highlighting distinct interpretative characteristics among the different visualization methods.

### 3.2. Classifier Model Development and Analysis

To reduce the dimensionality of the extracted features, this study applies PCA technology, which reduces the feature dimensions from 12,288 to 730, substantially improving computational efficiency. For further model development, we compared the performance of each base classifier (RF, SVM, KNN, XGB) with that of the ensemble stacking classifier. Table 3 summarizes the performance of each classifier across multiple evaluation metrics, including the AUC, accuracy, PPV, NPV, specificity, recall and F1 score, with bootstrap 95% confidence intervals. The stacking classifier achieved the highest point estimates for AUC and accuracy among the evaluated models, with an AUC of 0.921 and an accuracy of 0.871, while also showing a favorable F1-score of 0.727. Although the AUC and accuracy were relatively high, the lower F1-score reflects the threshold-dependent trade-off between precision and recall under class imbalance. Specifically, the stacking classifier achieved a PPV of 0.702 and a recall of 0.754, indicating that both false-positive and false-negative predictions remained. In addition, accuracy may be influenced by the predominance of non-RP slices, whereas AUC summarizes discriminative performance across multiple classification thresholds. Therefore, the F1-score and precision–recall results should be interpreted together with the AUC and accuracy rather than as contradictory findings. However, the performance differences should be interpreted together with the bootstrap confidence intervals and pairwise AUC comparisons.

Pairwise statistical comparisons among classifiers were further performed, and the corresponding p-values are provided in Supplementary Table S1. Because the complete pairwise comparison table was relatively large, it was provided as Supplementary Material to preserve the readability of the main performance table. Therefore, the numerical superiority of the stacking classifier should be interpreted together with the bootstrap 95% confidence intervals in Table 3 and the pairwise p-values in Supplementary Table S1.

**Figure 6:**
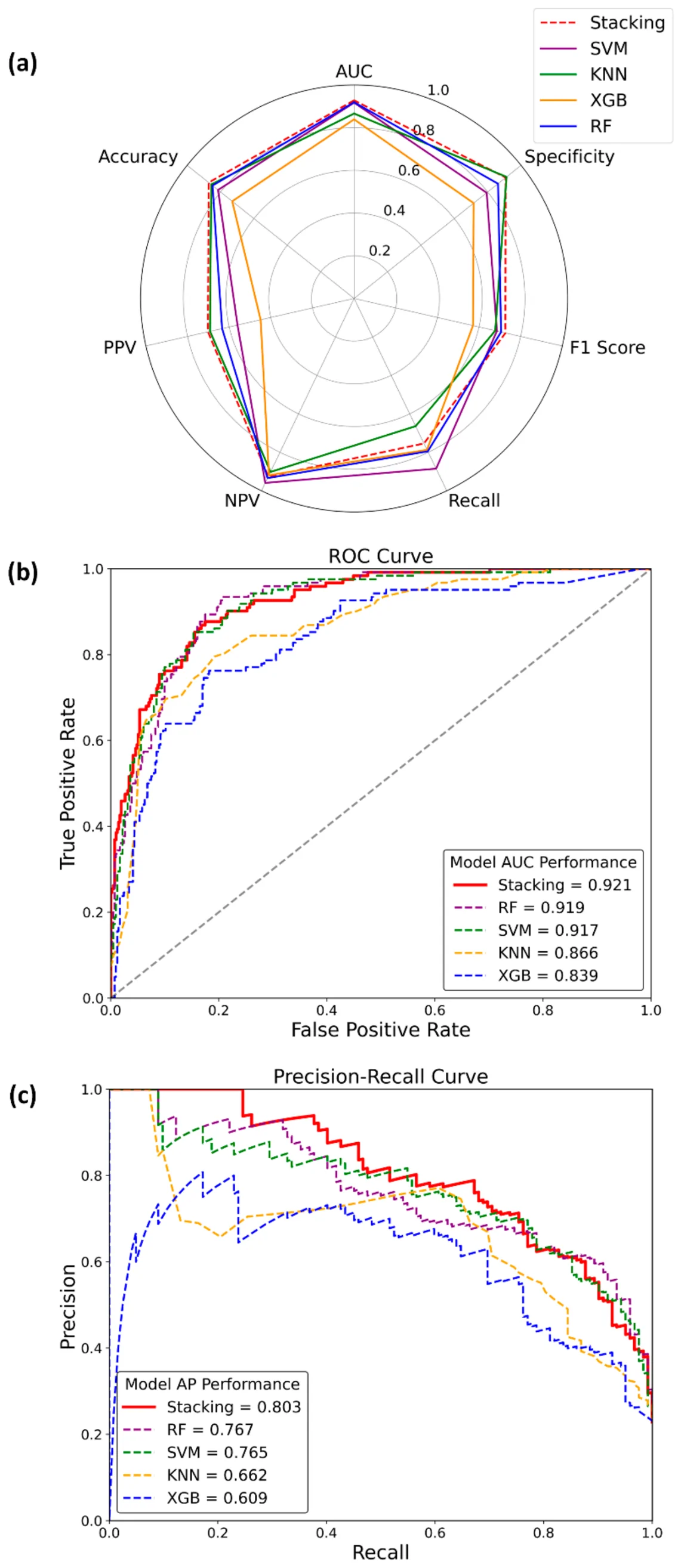
Visualization of the performance of each model, with model line colors as follows: Stacking classifier (red), SVM (purple), KNN (green), RF (yellow), XGB (blue). (**a**) Radar chart of evaluation metrics, (**b**) ROC curves, (**c**) Precision–Recall curves. Abbreviation: AUC: Area Under the ROC Curve, ROC: Receiver Operating Characteristic, AP: Average Precision, SVM: Support Vector Machine, KNN: K-Nearest Neighbor, RF: Random Forest, XGB: Extreme Gradient Boosting, PPV: Positive Predictive Value, NPV: Negative Predictive Value.

As shown in Figure 6a, the multiple evaluation metrics of the stacking classifier are close to the outer ring, indicating favorable overall performance. To visually display the performance differences among the evaluated models, we used a radar chart for visualization, which shows the overall performance patterns of the stacking classifier and the base classifiers. We used the AUC value (i.e., the area under the ROC curve) to assess the overall discriminative performance of the model. Figure 6b displays the ROC curves of the five models, among which the stacking classifier achieved an AUC of 0.921; Figure 6c shows the PR curves, with the stacking classifier achieving an AP of 0.803. This result provides additional information on the precision–recall trade-off for the RP-positive class under class imbalance. Overall, the stacking classifier showed favorable overall performance, although the magnitude and statistical significance of performance differences varied across individual comparisons.

To more directly evaluate the impact of PCA on model performance and computational efficiency, we compared the stacking classifier before and after PCA dimensionality reduction. Without PCA, the extracted feature dimension was 12,288, the accuracy was 0.873, and the training time was 49 min. After PCA, the feature dimension was reduced to 730, the accuracy was 0.871, and the training time decreased to 49 s. Therefore, PCA resulted in only a minimal decrease in accuracy of 0.002, while reducing the feature dimension by approximately 94.1% and shortening the training time by approximately 60-fold. These findings suggest that PCA provided a favorable trade-off by substantially improving computational efficiency while preserving comparable classification performance.

### 3.3. RP Computer-Assisted Image Review Assessment

On the basis of the trained stacking classifier model, we applied the model to CT images with RP features in the internal hold-out test set. The model identified RP-positive CT slices, and Grad-CAM-based visualization was used to provide qualitative visual cues for suspected RP-related regions. During data preprocessing, patient numbers and CT slice numbers were extracted from DICOM files and renamed for efficient processing. The trained stacking classifier was then applied to the image data in the test set, and samples classified as positive were reviewed qualitatively.

**Figure 7:**
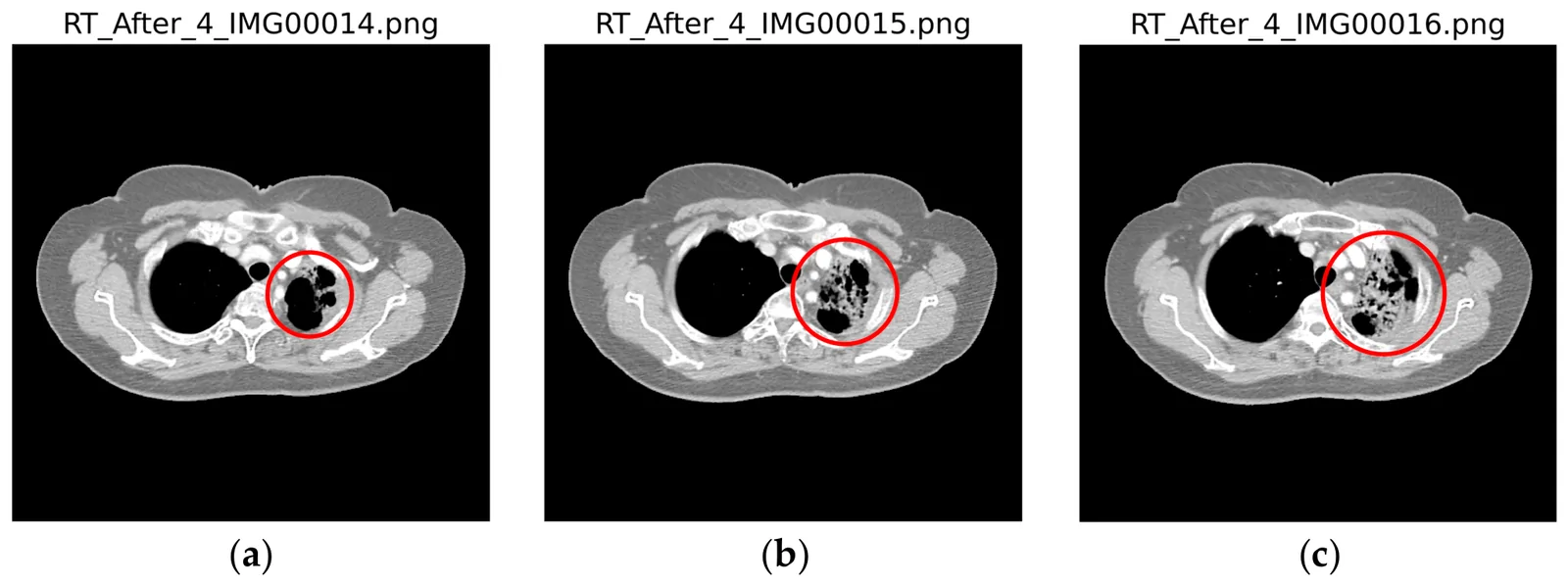
Representative RP-positive CT slices identified by the stacking classifier model. (**a**–**c**) are slices 14, 15, and 16, respectively, of patient No. 4. The red circles indicate suspected RP-related regions for qualitative visual reference. Abbreviation: CT: Computed Tomography.

As demonstrated in Figure 7, CT slices 14, 15, and 16 of patient No. 4 were classified as RP-positive and showed image findings visually consistent with suspected RP-related regions. The red circles indicate representative regions for visual reference. These findings should be interpreted as qualitative image-review support rather than quantitative evidence of precise lesion localization.

## 4. Discussion

The main finding of this study is that the RP-GAN model, developed by combining generative adversarial networks (GANs) with Grad-CAM technology, effectively extracts RP features from CT images and improves image-based classification performance through an ensemble learning model. Grad-CAM technology provided qualitative visual references to guide the selection of convolutional layers for feature extraction, and PCA was used for dimensionality reduction, which greatly improved computational efficiency by reducing the training time from 49 min to 49 s. Finally, image-based classification performance was improved using a stacking classifier, achieving an AUC of 0.921 and an accuracy of 0.871, with favorable performance compared with the individual base classifiers. The comparatively lower F1-score of 0.727 was primarily associated with the balance between PPV and recall in the imbalanced dataset, emphasizing the need to interpret threshold-dependent metrics together with AUC and precision–recall performance.

In terms of potential clinical image-review support, the framework may serve as an auxiliary tool for classifying RP-positive slices and providing model-generated qualitative imaging references. However, because this retrospective study did not measure physician review time, workflow efficiency, or missed-finding rates, the practical effect of these outputs on clinical image review remains to be evaluated prospectively. The model should therefore be regarded as an auxiliary image-review framework rather than a replacement for physician judgment.

In a practical clinical workflow, the proposed explainability output may serve as a second-reader aid during post-treatment CT image review. After CT slices are processed by the classifier, RP-positive slices can be flagged for closer inspection, and Grad-CAM heatmaps can provide visual cues indicating the image regions that contributed to the model prediction. These attention maps could potentially help clinicians prioritize suspicious slices and compare model-highlighted regions with clinical symptoms, radiotherapy history, and differential diagnostic considerations; however, this potential workflow benefit requires prospective evaluation. Importantly, the heatmaps are not intended to provide definitive lesion boundaries or replace physician interpretation. Instead, they provide qualitative image-based references that may improve transparency, support clinician confidence in model outputs, and identify cases in which model attention and clinical judgment are inconsistent and require further review.

A key advancement of this study is the development of a preliminary image-based auxiliary review framework that can identify RP-positive CT slices and highlight suspected RP-related regions in CT images by combining ensemble learning with Grad-CAM technology. As demonstrated in Figure 7, the proposed model not only classified CT slices as RP-positive but also provided visual cues corresponding to suspected RP-related image findings. The red circles indicate representative regions for visual reference. These outputs may support qualitative review of RP-related imaging findings, although their effect on clinical review efficiency has not yet been prospectively evaluated. They should be interpreted as qualitative attention-based findings rather than quantitative evidence of precise lesion localization.

Compared with conventional manual CT image assessment, which relies heavily on manual interpretation of CT images and is subject to variability on the basis of clinician experience, our method provides several potential advantages. Traditional approaches can be time-consuming and may miss subtle signs of RP due to human error or fatigue. In contrast, our AI-assisted framework may provide consistent support for clinical image review. Among the various visualization methods compared in this study, Grad-CAM was ultimately selected as the primary visualization tool because of its qualitative interpretability, computational simplicity, and widespread use in the literature. By combining multiple advanced techniques within a unified image-analysis workflow, our approach aims to balance image-based classification performance, computational efficiency, and interpretability. These attributes may improve the practical applicability of the proposed framework as an auxiliary image-review tool.

### 4.1. Impact and Comparison of Technological Choices

In this study, the applicable convolutional layers for RP feature extraction in the RP-GAN were selected with reference to Grad-CAM visualization. According to research by Selvaraju et al. [2], deeper convolutional layers capture high-level semantic information, which is consistent with our choice of conv2d_4 and conv2d_5 as feature extraction layers. This finding suggests that high-level image features extracted from deeper convolutional layers may contribute to RP image classification. Additionally, our stacking classifier achieved an accuracy of 0.871 for post-treatment CT-based RP image assessment, whereas Zhang et al. [9] reported an accuracy of 0.82 for RP prediction using CT and radiation dose images. However, these accuracy values should not be interpreted as a direct comparison of model performance because the two studies addressed fundamentally different clinical tasks. The present study focuses on assessing RP-related imaging findings that are already visible on post-treatment CT images, whereas Zhang et al. focused on predicting RP risk using CT and radiation dose information. The two studies also differed in input modalities, patient cohorts, labeling strategies, and evaluation settings. Therefore, the comparison in Table 4 is intended to illustrate methodological and clinical task differences rather than to demonstrate the superiority of one model over the other.

### 4.2. Advantages of Ensemble Learning Techniques

This study combined four machine learning models as base classifiers: RF, SVM, KNN, and XGBoost. Each classifier has strengths, such as RF’s ability to handle high-dimensional data and reduce overfitting risk [16], SVM’s excellent generalization ability in nonlinear classification problems [17], KNN’s effective handling of heterogeneous data [18], and XGBoost’s ability to progressively reduce errors by learning prediction residuals and its robustness to missing data [19]. The meta-classifier uses logistic regression (LR), a method that is simple to interpret, has strong noise resistance, and is suitable for integrating the predictive results of various base classifiers [20,21]. The combination of these classifiers through stacking techniques may enhance model stability and reduce the risk of relying on a single classifier. For example, Prakash et al. [22] combined 11 machine learning models for pediatric pneumonia diagnosis, and Rojas-López et al. [23] used eight models to diagnose spinal pathologies. Stacking techniques have also been applied in medical image classification tasks. These studies support the potential utility of stacking classifiers in integrating the advantages of different models. However, because the performance gain of the stacking classifier over some individual classifiers was modest in this study, future studies should further evaluate whether the added model complexity remains justified in larger datasets and external validation cohorts.

### 4.3. Application and Performance of PCA Dimensionality Reduction

After the CT image features were extracted, we used PCA technology for dimensionality reduction, effectively reducing the model training time. Particularly when the stacking classifier is used, the application of PCA substantially decreases the computational load, which is suitable for use in situations with limited hardware resources. In this study, PCA reduced the extracted feature dimensions from 12,288 to 730 and shortened the training time from 49 min to 49 s. Although the accuracy of the stacking classifier slightly decreased from 0.873 to 0.871 after PCA dimensionality reduction, the reduction in training time was substantial, suggesting that PCA provided a practical trade-off between computational efficiency and image-based classification performance. This dimensionality reduction method has shown excellent performance in other image classification studies, such as Aurna et al.’s [24] application in brain MR image classification, where PCA technology reduced the training time by six times, demonstrating its potential in various medical images.

In addition to computational efficiency considerations, PCA was selected in this study to maintain consistency within the overall model architecture. Since an unsupervised learning approach based on a GAN discriminator was adopted during the feature extraction phase, PCA, also an unsupervised method, was chosen for dimensionality reduction to ensure methodological coherence. Compared to fully connected layers that require additional training parameters, PCA provides rapid and stable dimensionality reduction without the need for defining extra learning objectives or adding structural complexity to the model. Nonetheless, we acknowledge that alternative dimensionality reduction techniques, such as fully connected layers or autoencoders, may offer different advantages. Future studies will consider incorporating various methods for comparison to further optimize model performance.

### 4.4. Study Limitations and Future Directions

Due to restrictions imposed by the scope of IRB approval, this study was limited to using data from a single medical institution. To ensure the objectivity of model evaluation, the dataset was partitioned at the patient level into a training set and an internal hold-out test set. Tenfold cross-validation was performed exclusively within the training set for stacking model development, whereas the internal hold-out test set was completely excluded from cross-validation, base-classifier training, generation of meta-level training features, and meta-classifier training, and was used only for final performance evaluation. Although this design enhances the reliability of the evaluation results, future studies will aim to obtain IRB approval from additional institutions in order to conduct multi-center external validation and further verify the model’s generalizability. Although this study included 2399 CT slices, these images were derived from only 46 patients; therefore, the effective clinical diversity of the dataset may be more limited than the slice-level sample size suggests. In particular, the number of non-RP patients was limited to 11, indicating that patient-level class imbalance remains an important limitation despite the use of model-level class weighting strategies. No a priori power calculation was performed, and the limited number of non-RP patients reduced the precision of the performance estimates and the ability to establish statistically robust differences among classifiers. Accordingly, the reported performance metrics and bootstrap confidence intervals should be interpreted as exploratory internal estimates rather than definitive evidence of statistical sufficiency. Therefore, the findings of this study should be interpreted as preliminary single-center evidence supporting the feasibility of the proposed RP-GAN framework, rather than definitive evidence of generalizability across broader clinical settings.

In addition to sample size and class imbalance, potential biases and confounding factors related to patient demographics, clinical characteristics, and CT acquisition procedures should also be considered. At the patient level, age, sex, BMI, tumor stage, and chemotherapy status may influence both the risk of RP and its CT imaging appearance. Although these baseline clinical characteristics are summarized in Table 1, they were used for descriptive purposes only and were not incorporated into the image-based model training process; therefore, residual confounding effects cannot be completely excluded. Regarding image acquisition, all data were collected from a single institution using the same CT scanner and institutional imaging workflow, and contrast-enhanced CT images were excluded. These measures helped reduce image heterogeneity related to scanner differences, acquisition protocols, and contrast administration. Furthermore, a consistent preprocessing pipeline, including Otsu-based segmentation, contour analysis, image centering, and pixel value standardization, was applied to reduce the influence of non-clinically relevant background structures and image-scale variability on model training. Nevertheless, the retrospective study design and the lack of external multi-center validation may still limit the generalizability of the model to other institutions, CT acquisition protocols, and clinical workflows. Future studies incorporating multi-center external validation and additional clinical and dosimetric variables are required to further assess and control potential biases and confounding factors.

Several practical challenges were also encountered during data collection and model development. The availability of eligible post-treatment CT images was limited by the retrospective study design, the scope of IRB approval, and patient privacy regulations. In addition, the limited number of non-RP patients introduced patient-level class imbalance, which may affect model stability despite the use of class weighting strategies. During model development, the image-based design also restricted the incorporation of clinical and dosimetric variables, such as dose-volume parameters, pulmonary function, smoking history, and detailed treatment-related factors. These practical constraints should be addressed in future studies with larger, multi-center, and more diverse datasets.

In this study, we applied class weighting during model training to mitigate class imbalance. However, future studies might adopt more advanced balancing strategies, such as sophisticated data augmentation techniques specifically designed to address imbalance [25], to further improve model stability and robustness [26]. Additionally, this study employed basic lung segmentation methods, including Otsu thresholding and contour analysis; however, implementing advanced segmentation algorithms [27] in subsequent research could potentially enhance lesion localization assessment and overall image-based classification performance. Because Otsu thresholding depends on grayscale intensity distributions and largest-contour selection, severe anatomical distortion or high-density surgical hardware may alter the generated mask and introduce preprocessing-related bias. Future studies should incorporate artifact-robust or deep learning-based thoracic segmentation methods and specifically evaluate segmentation consistency in patients with postoperative anatomical changes or implanted hardware.

The current study utilized 2D slices extracted from DICOM images based on the extended Lung-GAN architecture, which has demonstrated reliable and stable performance in extracting lung image features in prior research. Although 3D CT imaging inherently retains more spatial information and might theoretically enhance model performance, transitioning to a 3D architecture would require substantial reconstruction of the RP-GAN framework and substantially increase computational resource demands. Considering the scope and available resources of this study, we opted to validate our methodology using the established 2D architecture. Nevertheless, we recognize the potential advantages of employing a 3D model and plan to explore its effectiveness in future research.

To date, no relevant studies have applied the combined approach of GAN, Grad-CAM, PCA, and ensemble learning specifically to RP image assessment. Our integrated model addresses practical clinical demands by balancing image-based classification performance, interpretability, and computational efficiency. While the ensemble learning model demonstrated robust image-based classification performance in this study, it has not yet been directly benchmarked against state-of-the-art end-to-end deep learning methods, such as ResNet, DenseNet, EfficientNet, Vision Transformers, or medical foundation models. Future investigations should incorporate such comparisons to comprehensively evaluate and establish the relative merits and clinical applicability of our proposed approach.

Our proposed methodology integrates multiple modules, each serving distinct yet interdependent roles within the image-based classification framework: RP-GAN facilitates effective feature extraction, Grad-CAM enhances interpretability through visualization, PCA substantially reduces feature dimensionality to streamline computational demands, and ensemble learning optimizes classification performance. Due to their interlinked nature, independently conducting comprehensive ablation studies of each module was not feasible within the current scope. Nonetheless, selective comparative analyses were performed, such as evaluating the impact of PCA on training efficiency and classification accuracy. Results indicated that PCA substantially shortened training times with only a minimal decrease in classification performance.

Another methodological limitation is that the RP-GAN hyperparameters were adopted from the original Lung-GAN architecture and commonly used GAN training settings without systematic hyperparameter optimization. Because GAN training can be sensitive to the learning rate, batch size, optimizer parameters, and number of training epochs, the selected configuration may have influenced training convergence and the stability of the extracted feature representations. Therefore, the present findings reflect the performance of a specific hyperparameter configuration. Future studies should perform systematic hyperparameter optimization and repeated training experiments to evaluate the robustness and stability of the RP-GAN feature extraction process.

Although Grad-CAM heatmaps provided qualitative visual cues for suspected RP-related regions, this study did not include pixel-level clinician-annotated RP lesion masks or standardized region-of-interest annotations. During the revision process, we considered conducting a limited exploratory overlap analysis using a small subset of RP-positive test images. However, qualified clinical annotators with specific expertise in RP imaging were not available within the current study team or project resources, and the original retrospective study did not include a standardized expert-annotation workflow. Establishing clinically defensible reference masks would therefore require a separately organized annotation study, including the recruitment of appropriate clinical experts, development of a prespecified annotation protocol, blinded review of the original CT images with sufficient clinical and imaging context, and assessment of inter-observer agreement. These requirements could not be adequately established within the scope of the present study. We therefore did not use non-expert or ad hoc annotations solely to calculate exploratory overlap values, because such pseudo-reference masks could produce misleading estimates of spatial agreement. In addition, Grad-CAM heatmaps are attention-based explanatory maps rather than binary lesion segmentation outputs, and conversion of these heatmaps into binary masks would require a prespecified thresholding strategy that could substantially affect the resulting Dice coefficient, intersection over union, and hit-rate values. Accordingly, the Grad-CAM findings in this study should be interpreted only as qualitative image-based references rather than quantitatively validated evidence of lesion localization. Future studies should prospectively establish expert-annotated RP reference regions, blinded and standardized annotation procedures, predefined Grad-CAM thresholding strategies, and inter-observer agreement assessment before evaluating spatial consistency using the Dice coefficient, intersection over union, maximum-activation-point hit rate, or related localization metrics.

Another limitation is that the present model used post-treatment CT images alone; therefore, radiation dose distributions, dose-volume histogram (DVH) parameters, pulmonary function tests, chemotherapy history, smoking history, and other clinical variables were not incorporated. The absence of DVH parameters, such as the mean lung dose and the percentages of lung volume receiving specific dose levels, prevented the model from considering the quantitative dose burden associated with RP development. In addition, because DVH parameters do not retain spatial dose information, the absence of both DVH data and spatial dose distributions limited the ability to determine whether CT abnormalities were located within irradiated high-dose regions. Consequently, the model could not use dose–response consistency to distinguish radiation-induced pulmonary changes from other conditions with similar CT appearances, such as infection, tumor progression, pulmonary edema, or pre-existing fibrotic changes. Accordingly, the current model should be interpreted as an image-based auxiliary review framework rather than a comprehensive RP risk-prediction or differential-assessment model.

Future research will focus on several specific directions. First, larger and more diverse multi-center datasets will be collected to evaluate the robustness and generalizability of the proposed framework across different institutions, CT scanners, and CT acquisition protocols. Second, multimodal inputs, including dose-volume parameters, pulmonary function, smoking history, chemotherapy history, and other clinical variables, will be incorporated to develop a more comprehensive RP assessment model. Third, alternative model architectures, such as 3D CNNs, ResNet, DenseNet, EfficientNet, Vision Transformers, and medical foundation models, will be systematically compared with the current RP-GAN-based framework. Finally, quantitative validation of Grad-CAM heatmaps, including overlap analysis with radiologist-marked regions and layer-wise ablation analysis, will be performed to further assess the interpretability and clinical reliability of the model.

## 5. Conclusions

In this study, we developed a preliminary image-based auxiliary review framework for RP assessment by combining ensemble learning and Grad-CAM technology. The primary contribution of our research is the model’s ability to identify RP-positive CT slices and provide qualitative visual references for suspected RP-related regions. Because this retrospective study did not evaluate real-time clinical deployment or workflow turnaround time, these outputs should be interpreted as offline image-review references rather than evidence of immediate clinical availability. This capability may warrant prospective evaluation as a potential aid for reviewing suspected RP-related imaging findings.

Additionally, the integration of Grad-CAM technology enhances model interpretability by providing visual explanations that may help clinicians understand model attention. The application of PCA substantially reduced classifier training time under the reported experimental environment, demonstrating improved computational efficiency during model development.

In summary, this study provides an efficient and interpretable image-based auxiliary review framework for RP, with the major contribution being its ability to provide qualitative imaging references that may support RP image assessment. Prospective workflow studies are required to evaluate inference latency, total processing time, clinical system integration, and the effect of the framework on physician image-review time before any claim of real-time clinical use can be made. However, further multi-center external validation, quantitative localization assessment, and comparison with contemporary deep learning backbones or medical foundation models are required before broader clinical application as an auxiliary image-review tool.

## Figures and Tables

**Table 1:** Clinical Characteristics of Patients.

	Total	Without RP	With RP
	N = 46	N = 11 (23.9%)	N = 35 (76.1%)
**Age (years)**			
Range	43–90	47–88	43–90
(Mean ± SD)	67.30 ± 11.19	66.18 ± 13.30	67.66 ± 10.64
**Gender**			
Male	39 (84.8%)	10 (21.7%)	29 (63.1%)
Female	7 (15.2%)	1 (2.2%)	6 (13.0%)
**BMI (kg/m^2^)**			
Range	9.59–34.42	13.93–26.05	9.59–34.42
(Mean ± SD)	23.72 ± 4.72	21.02 ± 3.48	24.57 ± 4.78
**T stage**			
T1	8 (17.4%)	1 (2.2%)	7 (15.2%)
T2	15 (32.6%)	3 (6.5%)	12 (26.1%)
T3	11 (23.9%)	4 (8.7%)	7 (15.2%)
T4	12 (26.1%)	3 (6.5%)	9 (19.6%)
**N stage**			
N0	11 (23.9%)	1 (2.2%)	10 (21.7%)
N1	6 (13.0%)	3 (6.5%)	3 (6.5%)
N2	17 (37.0%)	5 (10.9%)	12 (26.1%)
N3	12 (26.1%)	2 (4.3%)	10 (21.8%)
**Chemotherapy**			
No	5 (10.9%)	1 (2.2%)	4 (8.7%)
Yes	41 (89.1%)	10 (21.7%)	31 (67.4%)

Abbreviation: RP: Radiation Pneumonitis, BMI: Body Mass Index.

**Table 2:** Dataset from KSVGH.

**Number of Patients**			
Class	Train set	Test set	Total
RP	28	7	35
Non-RP	8	3	11
**Number of Images**			
Class	Train set (%)	Test set (%)	Total
RP	420 (77.5)	122 (22.5)	542
Non-RP	1445 (77.8)	412 (22.2)	1857

Abbreviations: RP: Radiation Pneumonitis. Note: The training and test sets were divided at the patient level. All CT slices from the same patient were assigned exclusively to either the training set or the test set to avoid data leakage.

**Table 3:** Performance of the proposed stacking classifier and each base classifier with bootstrap 95% confidence intervals.

Classifier	AUC	Accuracy	PPV	NPV	Specificity	Recall	F1-Score
SVM	0.917 (0.889–0.939)	0.815 (0.775–0.841)	0.560 (0.488–0.620)	0.959 (0.934–0.976)	0.794 (0.753–0.829)	0.885 (0.822–0.933)	0.686 (0.628–0.742)
RF	0.919 (0.889–0.939)	0.848 (0.805–0.869)	0.634 (0.525–0.680)	0.934 (0.910–0.958)	0.862 (0.809–0.884)	0.795 (0.725–0.863)	0.706 (0.628–0.742)
KNN	0.866 (0.828–0.902)	0.856 (0.826–0.886)	0.692 (0.610–0.754)	0.902 (0.871–0.928)	0.913 (0.887–0.936)	0.664 (0.575–0.739)	0.678 (0.596–0.732)
XGB	0.839 (0.783–0.869)	0.730 (0.676–0.758)	0.449 (0.366–0.493)	0.919 (0.881–0.945)	0.716 (0.654–0.750)	0.787 (0.694–0.844)	0.571 (0.487–0.609)
**Stacking**	0.921 (0.893–0.943)	0.871 (0.845–0.899)	0.702 (0.620–0.777)	0.926 (0.904–0.952)	0.908 (0.874–0.932)	0.754 (0.684–0.833)	0.727 (0.667–0.779)

Abbreviation: AUC: Area Under the ROC Curve, ROC: Receiver Operating Characteristic, PPV: Positive Predictive Value, NPV: Negative Predictive Value, SVM: Support Vector Machine, RF: Random Forest, KNN: K-Nearest Neighbor, XGB: Extreme Gradient Boosting.

**Table 4:** Methodological comparison with previous RP-related studies.

Research	Task	Model	Reported Accuracy	Imaging Modality
This study	RP image assessment	RP-GAN	87.1%	CT images
Zhang et al. [9]	RP prediction	CT/RD-based deep learning model	82.0%	CT and radiation dose images

Abbreviation: RP-GAN: Radiation Pneumonitis Generative Adversarial Network, CT: Computed Tomography. Note: The reported accuracy values are provided for descriptive reference only and should not be directly compared because the studies differed in clinical objective, input modality, cohort characteristics, labeling strategy, and evaluation design.

## Data Availability

The source code for the RP-GAN model, including preprocessing, feature extraction, and stacking ensemble learning components, may be made available by the corresponding author upon reasonable request, subject to institutional approval and applicable data-use and research-governance requirements. Due to institutional review board (IRB) regulations and patient privacy considerations, the raw clinical and imaging data containing potentially identifiable information cannot be shared publicly. De-identified processed data sets (e.g., extracted feature matrices, trained model weights, and representative anonymized samples) that support the main findings of this study are available from the corresponding author upon reasonable request for research purposes and subject to institutional data use agreements and ethical approval. The shared processed data include RP and non-RP imaging samples derived from lung cancer patients with clinical characteristics summarized in Table 1, including age, sex, BMI, T/N stage, and chemotherapy status. However, because all data were collected from a single institution using a specific CT scanner model and institutional treatment workflow, the dataset may not fully represent broader patient populations, imaging protocols, or clinical environments. Therefore, the shared data should be interpreted as a single-center processed dataset suitable for reproducibility assessment and methodological verification, rather than as a fully generalizable multi-center benchmark dataset. A Zenodo record has been reserved for potential future archiving of the finalized code and de-identified processed data. Any public release will be subject to institutional approval, applicable ethical requirements, and final publication arrangements. The code used for our analyses will be available upon reasonable request from the corresponding author. Requests should be directed to T.F. Lee at tflee@nkust.edu.tw.

## Supplementary Materials

The following supporting information can be downloaded at: https://www.mdpi.com/article/10.3390/life16081269/s1.

## Abbreviations

**AI**	Artificial Intelligence
**AP**	Average Precision
**AUC**	Area Under the ROC Curve
**BMI**	Body Mass Index
**CNN**	Convolutional Neural Network
**CT**	Computed Tomography
**CUDA**	Compute Unified Device Architecture
**DICOM**	Digital Imaging and Communications in Medicine
**F1-score**	Harmonic mean of precision and recall
**GAN**	Generative Adversarial Network
**Grad-CAM**	Gradient-weighted Class Activation Mapping
**IRB**	Institutional Review Board
**KNN**	K-Nearest Neighbors
**LR**	Logistic Regression
**NPV**	Negative Predictive Value
**NSTC**	National Science and Technology Council
**PCA**	Principal Component Analysis
**PNG**	Portable Network Graphics
**PPV**	Positive Predictive Value
**PR**	Precision–Recall
**RF**	Random Forest
**ROC**	Receiver Operating Characteristic
**RP**	Radiation Pneumonitis
**RP-GAN**	Radiation Pneumonitis Generative Adversarial Network
**RTOG**	Radiation Therapy Oncology Group
**SVM**	Support Vector Machine
**VMAT**	Volumetric Modulated Arc Therapy
**XAI**	Explainable Artificial Intelligence
**XGB/XGBoost**	Extreme Gradient Boosting
