# Supplementary material for: Explainable AI for Image-Based Auxiliary Assessment of Radiation Pneumonitis on Post-Treatment CT Images Using GAN Grad-CAM and Ensemble Learning Techniques
Source: Life (Basel). 2026 Jul 31;16(8):1269. doi: 10.3390/life16081269 (PMC13514787; doi:10.3390/life16081269)
Supplement: Supplementary file 1 [file life-16-01269-s001.zip › life-4323742-supplementary.pdf]

Supplementary Table S1. The table presents the performance of the proposed stacking classifier and each base classifier on different evaluation metrics

Classifier	AUC	Accuracy	PPV	NPV	Specificity	Recall	F1-Score	*p-value*
SVM	0.917	0.815	0.560	0.959	0.794	0.885	0.686	0.460
RF	0.919	0.848	0.634	0.934	0.862	0.795	0.706	0.480
KNN	0.866	0.856	0.692	0.902	0.913	0.664	0.678	< 0.001
XGB	0.839	0.730	0.449	0.919	0.716	0.787	0.571	< 0.001
**Stacking**	0.921	0.871	0.702	0.926	0.908	0.754	0.727	

Abbreviation: AUC: Area Under the ROC Curve, ROC: Receiver Operating Characteristic, PPV: Positive Predictive Value, NPV: Negative Predictive Value, SVM: Support Vector Machine, RF: Random Forest, KNN: K Nearest Neighbor, XGB: Extreme Gradient Boosting

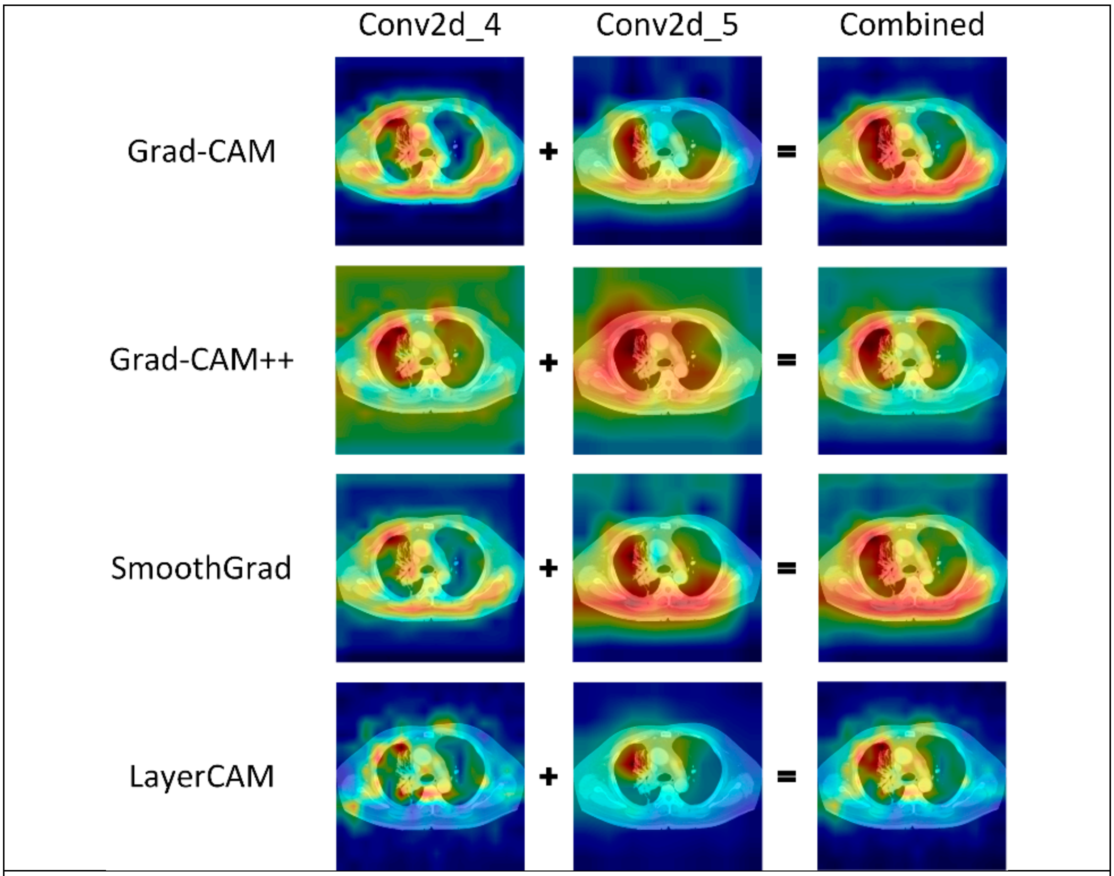

Supplementary Figure S1. Comparison of different visualization methods for highlighting the attention regions in RP images, demonstrating the effectiveness of Grad-CAM in accurately localizing key diagnostic areas.

Abbreviations: Grad-CAM: Gradient-weighted Class Activation Mapping
